# Positioning imatinib for pulmonary arterial hypertension: A phase I/II design comprising dose finding and single-arm efficacy

**DOI:** 10.1177/20458940211052823

**Published:** 2021-10-17

**Authors:** Martin R. Wilkins, Mikel A. Mckie, Martin Law, Andreas A. Roussakis, Lars Harbaum, Colin Church, J Gerry Coghlan, Robin Condliffe, Luke S Howard, David G Kiely, Jim Lordan, Alexander Rothman, Jay Suntharalingam, Mark Toshner, Stephen J Wort, Sofía S. Villar

**Affiliations:** 1 National Heart and Lung Institute, Faculty of Medicine, Imperial College London, Hammersmith Hospital, London, UK; 2 MRC Biostatistics Unit, School of Clinical Medicine, Cambridge Institute of Public Health, Cambridge, UK; 3 41444 Golden Jubilee National Hospital, NHS Greater Glasgow and Clyde, Clydebank, UK; 4 41444 Golden Jubilee National Hospital, University of Glasgow, Scotland, UK; 5 Royal Free Hospital, Royal Free London NHS Foundation Trust, London, UK; 6 Department of Infection, Immunity and Cardiovascular Disease, University of Sheffield, Sheffield, UK; 7 Sheffield Pulmonary Vascular Disease Unit, 7318 Sheffield Teaching Hospitals NHS Foundation Trust, Sheffield, UK; 8 National Pulmonary Hypertension Service, Hammersmith Hospital, Imperial College Healthcare NHS Trust, London, UK; 9 Newcastle Freeman Hospital, Freeman Road, High Heaton, Newcastle Upon Tyne, UK; 10 Royal United Hospital, Royal United Hospitals Bath NHS Foundation Trust, Bath, UK; 11 Heart Lung Research Institute, University of Cambridge, Cambridge, UK; 12 Department of Medicine, University of Cambridge, Cambridge, UK; 13 Royal Brompton Hospital, Guy’s and St Thomas’s Trust, London, UK

**Keywords:** Adaptive design, safety, tolerability, efficacy

## Abstract

Pulmonary arterial hypertension is an unmet clinical need. Imatinib, a tyrosine kinase inhibitor, 200 to 400 mg daily reduces pulmonary artery pressure and increases functional capacity in this patient group, but is generally poorly tolerated at the higher dose. We have designed an open-label, single-arm clinical study to investigate whether there is a tolerated dose of imatinib that can be better targeted to patients who will benefit. The study consists of two parts. Part 1 seeks to identify the best tolerated dose of Imatinib in the range from 100 and up to 400 mg using a Bayesian Continuous Reassessment Method. Part 2 will measure efficacy after 24 weeks treatment with the best tolerated dose using a Simon’s two-stage design. The primary efficacy endpoint is a binary variable. For patients with a baseline pulmonary vascular resistance (PVR) >1000 dynes · s · cm^−5^, success is defined by an absolute reduction in PVR of ≥300 dynes · s · cm^−5^ at 24 weeks. For patients with a baseline PVR ≤1000 dynes · s · cm^−5^, success is a 30% reduction in PVR at 24 weeks. PVR will also be evaluated as a continuous variable by genotype as an exploratory analysis. Evaluating the response to that dose by genotype may inform a prospective biomarker-driven study.

## Background and rationale

Pulmonary arterial hypertension (PAH) is an uncommon condition characterised by pre-capillary resistance to pulmonary blood flow in the absence of airway or parenchymal lung diseases, left heart failure or chronic thromboembolism.^1^^–3^ In around 50% of patients, there is no identifiable underlying cause and patients are classified as idiopathic PAH or, where there is a family history, heritable PAH. Histological examination of post-mortem or transplantation PAH lung tissue shows marked pulmonary arterial remodelling with vascular cell proliferation narrowing the vascular lumen.^4^

PAH is an unmet clinical need. Five-year mortality for idiopathic/heritable PAH managed by experienced centres in the UK is around 58% and long-term survival when associated with co-morbidities, such as connective tissue disease, is worse.^5^ The current licensed treatments (prostanoids, endothelin receptor antagonists, phosphodiesterase type 5 inhibitors and soluble guanylate cyclase stimulator) focus on pharmacologically manipulating three signalling pathways recognised for regulating vascular tone.^1^^–3^ These treatments have little impact on the underlying vascular remodelling^4^ and do not arrest or reverse the course of the condition.

The past decade has seen attempts to target other signalling pathways using drugs with pronounced antiproliferative or anti-inflammatory properties.^3,6^ One such drug is Imatinib, an orally active tyrosine kinase inhibitor.^7^^–10^ Imatinib inhibits platelet-derived growth factor receptor (PDGFR) alpha and beta, BCR-ABL, discoidin domain receptor (DDR) and c-KIT.^11^ Studies of lung tissue from PAH patients show increased PDGF activity and c-KIT expression associated with remodelled vessels.^12,13^ PDGF is a potent vascular smooth muscle cell mitogen,^14^ while c-kit is a marked of bone marrow–derived hematopoietic stem cells.^13^ Imatinib reverses pulmonary hypertension in experimental models.^7^ This is consistent with the antiproliferative properties of Imatinib, but it has also been reported that Imatinib can reduce pulmonary vascular tone.^15^^–17^

The most robust human data come from two placebo-controlled randomised clinical trials: a Phase II trial involving 59 patients^9^ and a Phase III trial (IMPRES) including 202 patients^10^ taking Imatinib 200 to 400 mg daily. The phase II study showed a significant decrease in pulmonary vascular resistance (PVR) compared to a placebo and IMPRES^10^ reported a significant improvement at 24 weeks in functional capacity, with a mean placebo-corrected treatment effect on six-minute walk distance (6MWD) of 32 m (95% confidence interval, 12–52; p = 0.002), and in PVR of –379 dyne · s · cm^–^^5^ (95% confidence interval, –502 to –255; p<0.001) in patients taking Imatinib along with their licensed vasodilatory treatments.^10^ Unfortunately, serious adverse events and discontinuations were more common in the Imatinib group^10^ and the FDA requested further safety data. The drug remains unlicensed for PAH.

Despite these concerns, and in the absence of alternative effective treatments, there remains considerable interest in the use of Imatinib for PAH, administered orally or by inhalation.^18^^–^^20^ Imatinib is established as a treatment for chronic myeloid leukaemia^21^ and gastro-intestinal stromal tumour.^22^ Imatinib is used occasionally in refractory PAH on compassionate grounds, but usually in doses lower than 400 mg, a dose used frequently in oncology. To address the previous safety concerns, and before moving on to an efficacy study, there is a need to establish a dose of Imatinib that is best tolerated by patients with PAH.

In positioning Imatinib for PAH, attention must also be given to inter-individual variability in response, seen with most drugs and evident with Imatinib.^10^ Where there are safety considerations, there is benefit in exploring if Imatinib can be better targeted at patients more likely to respond and reduce exposure to those least likely to benefit. Trough plasma levels are known to vary, influenced among other factors by substrates, inhibitors and inducers of CYP3A4.^23^ Variation in drug target expression and function may also be factors. For example, haematological malignancies driven by somatic *PDGFRB* mutations are very sensitive to Imatinib.^24,25^ Of note here, three separate population-based studies have identified a *cis*-acting protein quantitative trait locus (pQTL) located close to *PDGFRB*, influencing circulating levels of PDGFRB, that reaches genome-wide significance.^26^^–^^28^ If PDGFRB is an important target for Imatinib in PAH, common genetic variants influencing PDGFRB protein levels may affect therapeutic response, or at least the dose required to elicit a response, and the risk of dose-limiting adverse effects.

Here we detail the protocol for an adaptive clinical trial directed at identifying the best tolerated dose of Imatinib in PAH patients (Part 1) and discuss options for investigating the efficacy of that dose in a seamless follow-on study (Part 2), exploring response according to genotype. The protocol has been approved by the NHS Health Research Authority (IRAS PROJECT ID 274093, REC Reference 20/SC/0240).

## Objectives

The study has two main objectives, each studied in two distinct parts.

### Primary

Part 1: To identify the best tolerated dose of Imatinib (between 100 mg and 400 mg once daily for a target treatment duration of at least four weeks) in patients with PAH.

Part 2: To assess the efficacy of Imatinib administered at the best tolerated dose as identified in Part 1 (when taken once daily for 24 weeks) on PVR in PAH.

### Secondary

1. To analyse the change in PVR at 24 weeks according to genes that influence the actions of PDGF.

2. To identify plasma proteins and genetic variants that predict the clinical response to Imatinib.

3. To identify plasma proteins that report an early therapeutic response to Imatinib.

## Methods

### Overview

Part 1 follows a single-dose escalation protocol. We will recruit a minimum of 6 and up to 13 patients to explore four doses of Imatinib (100 mg, 200 mg, 300 mg and 400 mg once daily) with the aim of recommending a best tolerated dose using a Bayesian continual reassessment method (CRM).^29^ We consider the best tolerated dose to be that which has a 20% probability that a patient will not be able to continue imatinib for five consecutive days and we aim to identify the dose that is most likely to meet this tolerability criterion. For the CRM model, tolerability will be assessed after the first four weeks of treatment. Patients will, however, be continued on their allocated dose for up to 24 weeks to assess safety beyond the four-week tolerability evaluated by the CRM, with clinicians allowed to modify the dose in that period according to clinical need. This additional information on longer term tolerability will not influence the dose recommended by the CRM model for sequential patients in Part 1 but may be considered when making a final dose recommendation for Part 2 of the study.

Part 2 will measure the change in PVR from baseline after 24 weeks of Imatinib at the best tolerated dose using a Simon two-stage design.^30^ For the purpose of identifying ‘responders’, we define treatment response to be an absolute reduction in PVR of ≥300 dynes · s · cm^−5^ at 24 weeks for patients with a baseline PVR >1000 dynes · s · cm^−5^ and a 30% reduction in PVR at 24 weeks for patients with a baseline PVR ≤1000 dynes · s · cm^−5^. Define *p* as the true response rate of the treatment. Our null hypothesis is H_0_: *p* ≤ *p*_0_ = 0.1 and alternative hypothesis is H_1_: *p* > *p*_0_ = 0.1. We denote α the type-I error-rate, defined here the probability of rejecting the null hypothesis for a treatment that has response rate *p*_0_. We define β as the type-II error-rate and therefore 1–β as the power; here the probability of rejecting the null hypothesis for a treatment that has response rate *p*_1_. We choose *p*_1_ = 0.27. We will choose a design that has a type-I error-rate of α ≤ 0.05 and power 1–β ≥ 0.8, with a maximum sample size of 34. One suitable Simon design includes an interim analysis once 13 patients have completed 24 weeks of treatment on the recommended best tolerated dose. If there are zero responses or one response in these 13 patients, the study will be stopped without rejecting the null hypothesis. Otherwise, that is, if there are two or more responses, additional patients will be recruited for a total of 34 evaluable patients. In such a design, the null hypothesis is rejected if seven or more responses are observed in the 34 patient cohort. This design and its operating characteristics are described in the statistics and data analysis plan section.

### Study population

The study will recruit patients with symptomatic PAH. A documented diagnosis of PAH by right heart catheterisation is required prior to screening with evidence of a resting mean pulmonary artery pressure ≥25 mmHg, pulmonary capillary wedge pressure ≤15 mmHg, PVR >400 dynes · s · cm^–^^5^ (>5 wood units) and normal or reduced cardiac output. Brain imaging is required at baseline to exclude prior clinically significant cerebral haemorrhage. The inclusion and exclusion criteria are given in Table 1. Patients will be recruited from specialist centres recognised for their expertise in the management of PAH (e.g., https://digital.nhs.uk/data-and-information/clinical-audits-and-registries/national-pulmonary-hypertension-audit#audit-reports) and the UK PAH cohort study (https://ipahcohort.com). Given the low prevalence of PAH, conducting a trial of this size for this condition without this support would be challenging.

**Table 1.** Summary of entry criteria.

*Inclusion criteria*:
1. Subjects aged between 18 and 80 years old.
2. PAH which is idiopathic, heritable, associated with connective tissue disease, PAH after ≥? 1 year repair of congenital systemic to pulmonary shunt, or PAH associated with anorexigens or other drug.
3. Subjects willing to be genotyped for genes that influence PDGF activity.
4. Resting mean pulmonary artery pressure ≥25 mmHg, pulmonary capillary wedge pressure ≤15 mmHg, PVR > 400 dynes · s · cm^−5^ (5 wood units), and normal or reduced cardiac output.
5. Six-minute walking distance >50 m at entry.
6. Stable on an unchanged PAH therapeutic regime comprising at least 2 therapies licensed for PAH (any combination of endothelin receptor antagonist, phosphodiesterase inhibitor or prostacyclin analogue) for at least one month prior to screening.
7. Able to provide written informed consent prior to any study mandated procedures.
8. Contraception: Fertile females (women of childbearing potential) are eligible to participate after a negative highly sensitive pregnancy test, if they are taking a highly effective method of contraception during treatment and until the end of relevant systemic exposure. Fertile males who make use of condom and contraception methods during treatment and until the end of relevant systemic exposure in women of childbearing potential.
*Exclusion criteria:*
1. Unable to provide informed consent and/or are non-fluent speakers of the English language.
2. Hypersensitivity to Imatinib or to any of the excipients.
3. Clinically-significant renal disease (confirmed by creatinine clearance <30 ml/min per 1.73 m^2^).
4. Clinically-significant liver disease (confirmed by serum transaminases >3 times than upper normal limit).
5. Patients receiving oral and/or parenteral anticoagulants.^a^
6. Anaemia confirmed by haemoglobin concentration <10 g/dl.
7. History of thrombocytopenia.
8. Individuals known to have haemoglobinopathy sickle cell disease, thalassaemia.
9. Hospital admission related to PAH or change in PAH therapy within 3 months prior to screening.
10. History of left-sided heart disease and/or clinically significant cardiac disease, including but not limited to any of the following:
a. Aortic or mitral valve disease (stenosis or regurgitation) defined as greater than mild aortic insufficiency, mild aortic stenosis, mild mitral stenosis, moderate mitral regurgitation.
b. Mechanical or bioprosthetic cardiac valve.
c. Pericardial constriction, effusion with tamponade physiology, or abnormal left atrial size.
d. Restrictive or congestive cardiomyopathy.
e. Left ventricular ejection fraction ≤50% (measured in echocardiogram at screening).
f. Symptomatic coronary disease.
g. Significant (2+ for regurgitation) valvular disease other than tricuspid or pulmonary regurgitation.
h. Acutely decompensated left heart failure within 1 month of screening
i. History of untreated obstructive sleep apnoea
11. Evidence of significant lung disease on high-resolution CT (if available) or recent (performed within 12 months) lung function, where FEV1 < 50% predicted and FVC < 70% predicted, and DLCO (or TLCO) < 50% predicted if any CT abnormalities; judged by the Site Physician.
12. Patients with a history of uncontrolled systemic hypertension.
13. Acute infection (including eye, dental, and skin infections).
14. Chronic inflammatory disease including HIV, and Hepatitis B.
15. Women of childbearing potential who are pregnant or breastfeeding (if applicable).
16. Previous intracerebral haemorrhage.
17. Patients who have received an Investigational Medicinal Product (IMP) within 5 half-lives of the last dose of the IMP or one month (whichever is greater) before the b.

^a^This does not apply to single antiplatelet therapy.

Some patients with PAH who meet the entry criteria will have implanted FDA/CE-approved pulmonary artery pressure monitors (CardioMEMS^TM^) and cardiac rhythm monitors that capture and relay cardiopulmonary haemodynamics as well as daily activity to their local hospital. These patients also have a remote monitoring system to capture daily blood pressure, oxygen saturations and body weight. This permits regular monitoring at home. By including some patients with CardioMEMS^TM^ in Part 1 of this study we expect to obtain additional data on the effect of Imatinib to support safety monitoring, the timing and magnitude of any change in pulmonary haemodynamics and the relationship of plasma Imatinib levels to pulmonary haemodynamics that could inform the design of Part 2 of this study. We will present baseline characteristics and results of this subgroup of participants, without formal hypothesis testing, to explore any differences between instrumented and non-instrumented patients.

### Investigational product

Imatinib mesilate 100 mg or 400 mg tablets.

## Outcome measures and data collection

A tabulated summary of all visits and assessments is provided in Appendix 1.

### Primary outcome measure

Part 1: Discontinuation of the drug for more than five consecutive days due to Grade 2 or above Adverse Events defined by the National Cancer Institute (NCI) Common Terminology Criteria for Adverse Events (version 5.0, 2017), adapted for this study. For example, if a patient experiences an adverse event and stops taking the study drug for two days but recovers and completes the four-week treatment after the treatment break, the dose will be considered tolerable. However, if the treatment break is longer than five days the dose will be considered intolerable. In all cases, all adverse events will be recorded and reported irrespective if treatment is tolerated.

**Fig. 1.**
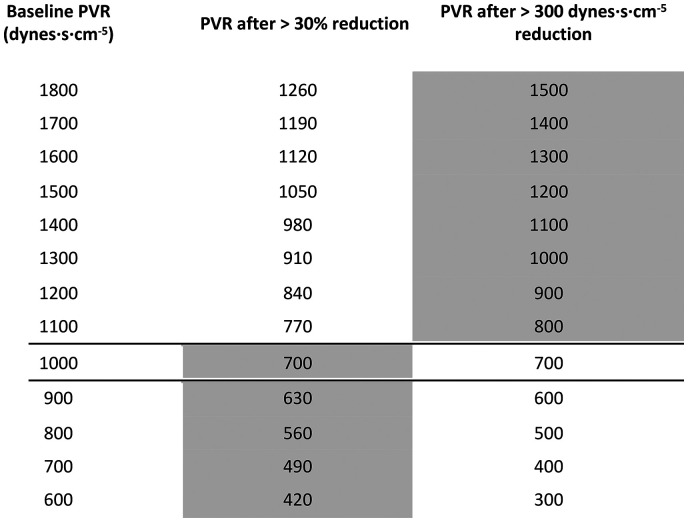
Change in pulmonary vascular resistance (PVR) relative to baseline based on % and absolute values. Above a baseline of 1000 dynes, an absolute reduction of 300 dynes is clinically meaningful, while with a baseline of 1000 dynes and below, a 30% reduction in PVR is deemed to be clinically significant.

Part 2: The primary efficacy endpoint is a binary variable. For patients with a baseline PVR >1000 dynes · s · cm^−5^, an absolute reduction in PVR of ≥300 dynes · s · cm^−5^ at 24 weeks is clinically meaningful. This was the mean reduction recorded in the Phase II Imatinib study in which the baseline PVR was 1124 dynes · s · cm^−5^.^9^ Given that this absolute change is a substantial fall in PVR for patients with a baseline below ≤1000 dynes · s · cm^−5^,^9^ for these patients, proportionate success is a 30% reduction in PVR at 24 weeks.^31,32^ Fig. 1 demonstrates how this translates into an equally meaningful reduction in PVR relative to baseline.

### Secondary measures

These comprise change from baseline to 24 weeks in (a) 6MWD from baseline, a measure of functional capacity, (b) right ventricular ejection fraction by echocardiogram, (c) plasma NT-proBNP levels, (d) quality of life score and (e) PVR according to genes that regulate PDGF activity.

### Exploratory outcome measures

Blood samples will be taken for Imatinib levels for pharmacokinetic analysis and for proteomic analysis. Pharmacokinetic data will be analysed with respect to tolerability and pharmacodynamic response. Plasma protein levels will be investigated for a profile that predicts response to Imatinib and/or provides an early (four week post-starting the drug) signal predicting response.

## Statistics and data analysis plan

**Fig. 2.**
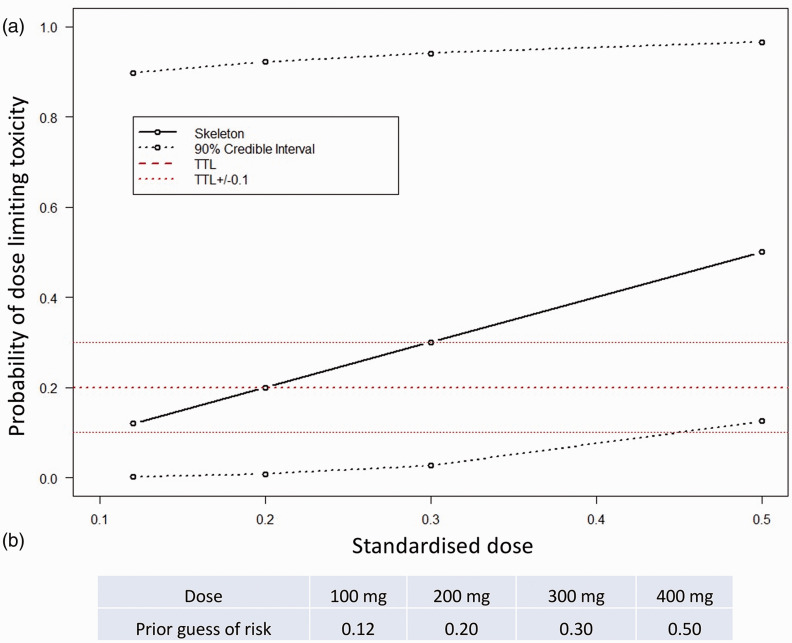
(a) Prior dose non-tolerability skeleton with (b) prior guesses of probability of intolerable response.

In Part 1, the dose selection of Imatinib for patients will be guided using a Bayesian CRM, allowing us to include previous experience with the study drug to best understand tolerability in an efficient and sequential manner. An initial dose/toxicity skeleton was generated based on a one-parameter power model in the form, *p*(tox|*d*) = *d*^α^, where *d* is the standardised dose and our single parameter α follows a gamma (1,1) distribution. Taken together with our prior belief of toxicities based on expert opinion and data from the IMPRES trial,^10^ we built our initial skeleton dose toxicity curve (Fig. 2).

**Fig. 3.**
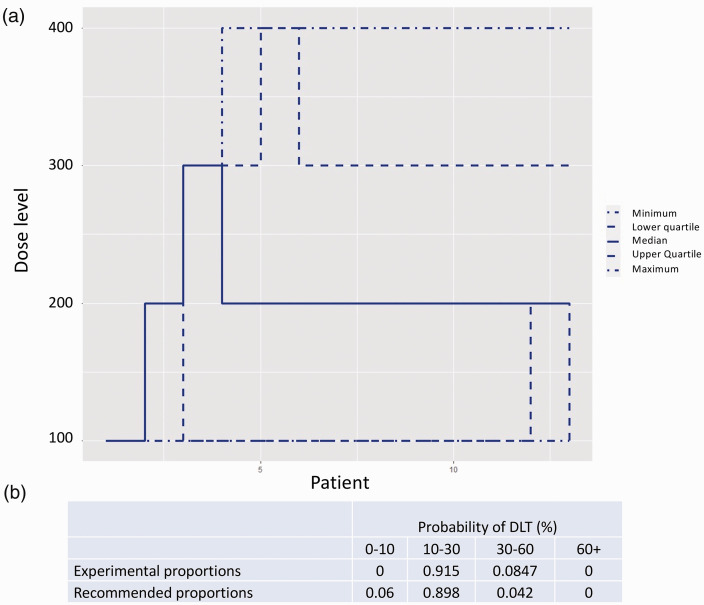
Simulation results based on the trial design outlined in Part 1. (a) Simulated trial trajectories based on 2000 simulations and our prior skeleton. (b) A table showing the average dose recommendations of 2000 simulations based on our prior understanding of tolerability being true.

Patients will be recruited into the trial sequentially; the first patient will receive the lowest dose of Imatinib (proposed starting dose of 100 mg). The best tolerated dose will be re-estimated after each patient completes the four-week follow-up, or earlier if a patient experiences an intolerable response prior to the four-week follow-up, and this will be recommended as the dose for the subsequent patient. The dose escalation scheme is limited to a single 100 mg dose change with no dose skipping. A safety stopping rule has also been imposed where, if the model identifies with 90% certainty that the lowest dose has a higher non-tolerability probability than the target non-tolerability level (TNL) of 20%, the study will stop. The minimum and maximum number of patients to enter Part 1 of the trial will be 6 and 13, respectively. We included a lower bound of recruitment at six patients to ensure that any intolerable responses in the first patients do not trigger early termination without adequate exploration of the doses. Under these constraints, using simulation studies, we have explored the operating characteristics of this trial design. If our prior tolerability expectations hold true, a simulation study has shown the trial design will correctly predict a dose within 10% of the TNL 90% of the time (Fig. 3).

After the 13th patient completes follow-up, tolerability and 90% credible intervals will be calculated for each dose level to generate the dose/tolerability quotient based on our four-week follow-up data and our prior understanding. We aim to reach a precision of 20%. If it is not reached after the first 13 patients, we are able to recruit further patients until we reach a suitable level of precision. We will also report Serious Adverse Events and toxicity incidence with or without withdrawal from study.

The dose that will be recommended for Part 2 of the study will be decided using the four-week tolerability data derived using the CRM in Part 1, in conjunction with longer term safety and efficacy data. As patients will continue their allocated dose after their four -week treatment, the sequential nature of Part 1 will allow us to have access to long-term safety data. In the best case at the point of the final patient four-week follow-up, all other patients will have been on the study drug for at least eight weeks. Using these data, we will ensure that the longer term safety does not deviate from the formally measured tolerability measured after four weeks. This is an important validation step for Part 2 as the treatment period is longer than Part 1. In the worst case that all patients do not continue treatment after four weeks we will recommend a dose based on the four-week data and any potential efficacy data from the patients in Part 1 that have implanted cardiac pressure instruments.

In Part 2, the efficacy of the best tolerated dose determined in Part 1 will be estimated in a single-arm, open-label trial. The trial will contain at least one interim analysis, at which point the trial may be stopped early for futility only (freeing resources for other treatments of this rare condition). The trial will continue to recruit even if final trial success is guaranteed, to enable data to be collected for additional analyses. In particular, we would like to maximise the data obtained for the analysis of response by genotype. The required operating characteristics for the trial are a type-I error-rate of 0.05 for a response rate *p* = *p_0_* = 0.10 and a power of 80% for a response rate *p* = *p_1_* = 0.27.

**Fig. 4.**
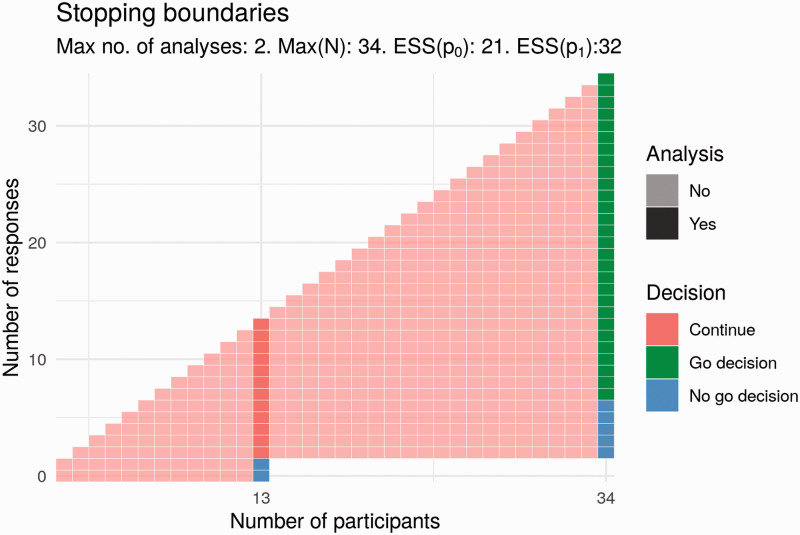
Potential Simon design for Part 2.

The simplest design approach for such a trial is a single-stage trial, where a fixed number of participants, *N*, receive the treatment. In a single-stage trial, success is declared if the final number of responses, *r*, exceeds some specified value. For the design parameters and required operating characteristics of this trial, the smallest possible sample size for a single-stage trial is *N = 32*. A popular alternative to the single-stage design is the Simon design.^30^ The Simon design is a two-stage design, meaning that there is an interim analysis in addition to the final analysis. This interim analysis takes place when some *n_1_* results are available. At this interim analysis, the number of responses is compared to a specified value, *r_1_*. If the number of responses does not exceed the specified interim value of *r_1_*, the trial will be stopped with no further recruitment. Otherwise, the trial continues to recruit until some maximum sample size, *N*, is reached. The advantage of the Simon design over a single-stage design is that when the treatment does not show promise, a decision is reached more quickly, without an increase in type-I error-rate. Fig. 4 shows an example of a Simon design that satisfies the required type-I error-rate and power. In this trial, an interim analysis takes place after *n_1_* = 13 results are available. At this point, the trial would end with a ‘no go decision’ if the number of responses is less than or equal to *r_1_* = 1. Otherwise, the trial continues until all *N* = 34 results are available, at which point a ‘go decision’ would be made or trial success declared if the number of responses observed is greater than *r = *6. If the number of responses observed is less than or equal to 6, a no-go decision would be made.

**Fig. 5.**
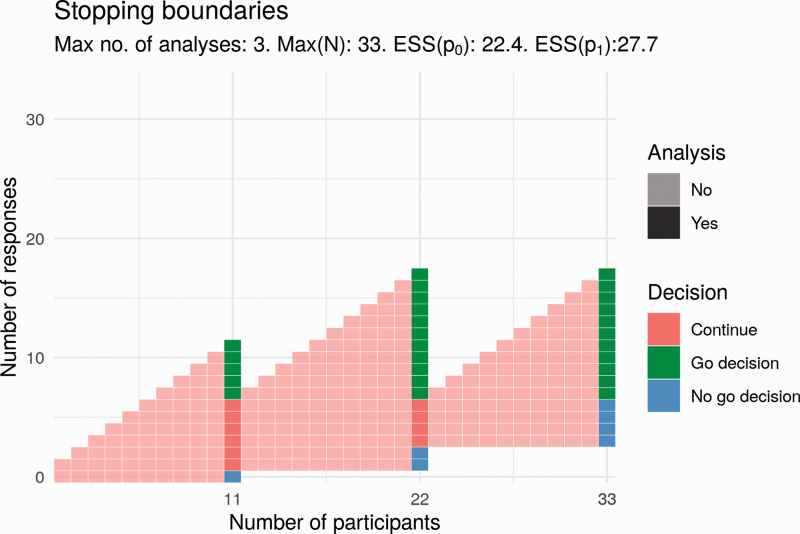
Potential design for Part 2 that uses stochastic curtailment.

We have modelled other adaptive designs satisfying the same operating characteristics. In the Simon design, early stopping may only occur at the interim analysis, that is, stopping is not permitted even when the final trial decision, go or no go, is known with certainty. For example, this would be the case in the above Simon design after 2 responses out of 30 results, at which point a no-go decision is certain even if all remaining results are responses. Stopping the study under this design when such certainty exists is known as **non-stochastic curtailment**. It may be the case that a go decision is extremely likely or extremely unlikely, though not yet certain. For example, this is the case in the above Simon design after 2 responses out of 29 results, where a go decision is still possible but unlikely even if the treatment has the desired response rate. In this example, 5 responses would be required from the 5 remaining results in order to reach 7 responses in total (and thus a go decision). Stopping a trial in such circumstances, when a go decision is particularly likely or unlikely, is known as **stochastic curtailment**. Permitting curtailment of either kind in a trial design can result in a design with more appealing properties than an equivalent Simon design. In particular, designs using curtailment may be superior in terms of a combination of the following three criteria: maximum sample size *N*, expected sample size if response rate *p* = *p_0_* = 0.1 (ESS(*p_0_*)), that is, if the treatment does not work and expected sample size if response rate *p* = *p_1_* = 0.27 (ESS(*p_1_*)), that is, if the treatment works.^33,34^ One example of such a design is shown in Fig. 5.

Compared to the Simon design, the proposal in Fig. 5 has a smaller maximum sample size (33 vs. 34) and a smaller expected sample size if the treatment works (27.7 vs. 32). The expected sample size if the treatment does not work is greater (22.4 vs. 21). There is one additional analysis; these interim analyses would take place after 11 and 22 participants. Here, the trial would end early for lack of effect if there are 0 responses at the first analysis or if there are fewer than 3 responses at the second analysis. This trial would end for a positive effect only if trial success by the end of the trial was certain; that is, the number of responses is greater than 6.

We have the option to change the design of Part 2 by amendment. We will inform the decision on the design by making use of tolerability and efficacy data collected in Part I. We will undertake two sensitivity analyses for the primary outcome in Part 2: we will compare response rates between participants with PVR > 1000 dynes · s · cm^−5^ and PVR ≤1000 dynes · s · cm^−5^ at baseline. We will also analyse the change in PVR from baseline as a continuous outcome. The design realisation used, that is, the specific set of interim analysis points and corresponding stopping boundaries, will be chosen before the beginning of Part 2. While incorporating, for example, non-stochastic curtailment for futility would be useful as it would improve the operating characteristics of the trial, stopping this trial has to be weighed against the possibility that it would limit the opportunity to explore a genotype–phenotype relationship. We reiterate that the primary analysis is fixed and independent of the design: the null hypothesis is that the probability of response is p_0_ = 0.1, and this will only be rejected if the number of responses is greater than some specified number *r*.

## Discussion

The two main aims of this study are (a) to identify a best tolerated dose of Imatinib for patients with PAH and (b) to establish its efficacy at this dose. An exploratory aim is to evaluate the response to Imatinib by patient genotype.

The dose range that will be explored, 100 mg to 400 mg daily, is based on IMPRES and clinical experience with the drug outside that study. In IMPRES, patients who were able to continue Imatinib for six months showed an improvement in 6MWD and haemodynamic measurements, but only 47% of patients were able to tolerate the drug at a dose of 400 mg for at least 11 weeks^10^; and practically all patients discontinued the drug in the long-term extension.^35^ Anecdotal reports suggest that lower doses, as low as 100 mg daily, used on compassionate grounds are better tolerated and may still offer patient benefit.

Our decision to opt for a Bayesian CRM in Part 1 means we can take advantage of prior clinical experience of using the drug. Integrated with data accrued during the proposed study, we increase our chances of correctly identifying the best tolerated dose with fewer patients than otherwise possible with other dose-finding strategies. It is appreciated that this has to be balanced against the time delay imposed by the sequential recruitment of patients at a minimum of four week intervals, the census point for assessing tolerability for the statistical model, and the additional time needed to update the statistical model and assemble the data safety monitoring team to sign off the dose for each patient. The decision to use four weeks as the census point is a compromise. Experience from IMPRES suggests that the majority of patients that discontinue Imatinib do so in the first eight weeks.^10^ Leaving a minimum of eight weeks between decisions would prolong Part 1 but as the first patients to be recruited will be followed up while new patients are enrolled, we will not lose information from later clinical presentations of poor tolerability. These later developments will not inform the statistical model for dose recommendation in Part 1, but later tolerability and safety concerns will be factored into the discussion around the choice of dose for Part 2.

The inclusion of patients with CardioMEMS^TM^ provides additional opportunities. Here, daily PAP measurements will be available to provide insight into the haemodynamic response to the drug. Interindividual differences in clinical response to Imatinib are well documented, with some patients benefiting substantially from addition of the drug to existing therapy.^8,18,19^ A responder analysis of patients in IMPRES shows that some patients gained a >60 m increase in 6MWD and/or showed substantial reductions (>30%) in PVR.^10^ In part, this may be due to variability in exposure to the drug; plasma tough levels at a given dose are influenced by body weight, haemoglobin concentration and concomitant therapies such as sildenafil and endothelin antagonists.^23,36^ Detailed steady-state pharmacokinetic studies in patients with CardioMEMS^TM^, measuring both free and total plasma levels, will permit pharmacokinetic:pharmacodynamic analysis to better understand the need to individualise dose to optimise plasma levels. The time-course of response of PAP to Imatinib exposure will also inform the duration of Part 2, currently set at 24 weeks but with the option to shorten if patients show a clear response in a shorter timeframe.

Part 2 of PIPAH builds on experience with the best tolerated dose from Part 1. Here, the use of a multi-stage design, either a two-stage Simon design or a multi-stage design using stochastic curtailment,^33,34^ permits an early decision on futility and/or efficacy. Such designs reduce the number of participants receiving an inefficacious treatment and allow investigators to consider other treatments sooner, while increasing the speed with which an efficacious treatment can be identified and the next phase planned. The type-I error-rate and power are controlled. While we are primarily concerned with making a decision regarding whether or not to continue investigating Imatinib, estimation of the response rate is also of interest. We acknowledge the possibility of biased estimation when stopping a trial early and would account for this by using an unbiased estimator. We also acknowledge the possibility of delayed responses increasing actual sample size compared to planned sample size. We have considered curtailment designs with other properties that may be valuable; for example, permitting early stopping for lack of effect only, or permitting early stopping only after a certain level of information is available. All considered, we have opted for the Simon two-stage design at this point to enable an exploratory analysis based on genotype; if the study is positive but stopped early, we would have fewer patients to conduct the genotype–phenotype analysis.

A pre-defined genotype-response of interest in Part 2 takes advantage of recognised genetic variation associated with *PDGFRB*. In two studies of 3301 and 5368 individuals, respectively, the same sentinel variant (rs2304058) was detected with an effect on circulating PDGFRB levels; each minor allele was associated with one standard deviation in plasma PDGFRB levels.^27,28^ The effect of this common variant on the circulating proteins levels was independent of protein-altering, germ-line variants in *PDGFRB.*^27^ PDGFRB expression is upregulated in lung tissue from PAH patients^12^ and may be involved in the pharmacological effect of Imatinib on pulmonary vascular tissue; the IC_50_ of Imatinib for PDGFRB is around 600 nM,^37^ a concentration exceeded by trough plasma levels (averaging 700 ng.ml^−1^) in IMPRES.^10,23^ Patients entering PIPAH will be genotyped for the rs2304058 *cis*-pQTL variant. DNA will also be available for additional genotype-response analysis and these data will be used to inform a follow-on biomarker-driven study if appropriate. Analysis of plasma samples taken at baseline and after starting Imatinib will permit analysis for circulating biomarkers that might predict response or signal early a likely positive effect of the drug on PVR.

In summary, we present a protocol to evaluate and position Imatinib as a treatment for PAH based on the use of adaptive designs that offer potential for efficiencies over more traditional designs. In Part 1, we employ CRM to assess tolerability and inform a dose for an efficacy study. In Part 2, we introduce and discuss the merits of Simons two stage and curtailment designs to enable early stopping for lack of effect. We anticipate that including some patients with CardioMEMS^TM^ in Part 1 we will gain some insight into how to optimise Part 2.

## Appendix 1. Schedule of events

**Table:**

Study period	Pretreatment	Dosing	Open label treatment period									Follow-up	
Assessment name	Assessment 1 (screening)	Assessment 2 (baseline)	Telephone assessment	Telephone assessment	Telephone assessment	Assessment 3	Assessment 4	Assessment 5	Telephone assessment	Telephone assessment	Assessment 6	Telephone assessment	Unscheduled assessment
Location	Clinic	Clinic/Home	(	(	(	Clinic/Home	Clinic/Home	Clinic/Home	(	(	Clinic/Home	(	Clinic/Home
Time (weeks)	Before Week 0	Week 0	Week 1	Week 2	Week 3	Week 4	Week 8	Week 12	Week 16	Week 20	Week 24	Week 28^g^	when needed
Assessment window (days)	0–28	± 3 days	± 3 days	± 3 days	± 3 days	± 3 days	± 3 days	± 3 days	± 3 days	± 3 days	± 3 days	± 3 days	n/a
Inclusion/exclusion criteria	X	–	–	–	–	–	–	–	–	–	–	–	–
Written informed consent	X	–	–	–	–	–	–	–	–	–	–	–	–
Demographics	X	–	–	–	–	–	–	–	–	–	–	–	–
Medical and medication history	X	–	–	–	–	–	–	–	–	–	–	–	–
Physical examination	X	X	–	–	–	X	X	X	–	–	X	–	X
Concomitant medications	X	X	X	X	X	X	X	X	X	X	X	X	X
Vital signs	X	X	–	–	–	X	X	X	–	–	X	–	X
WHO functional class	X	–	–	–	–	X	X	X	–	–	X	–	X^e^
Six-minute walk test (6MWT)	X	X	–	–	–	X	X	X	–	–	X	–	X^e^
Borg dyspnoea index	X	X	–	–	–	X	X	X	–	–	X	–	X^e^
*Right heart catheterization	–	X^a^^,h^	–	–	–	–	–	–	–	–	X^a^	–	–
Mouth swab sample	X	–	–	–	–	–	–	–	–	–	–	–	–
Haematology blood tests	X	X	–	–	–	X	X	X	–	–	X	–	X^e^
Clinical chemistry tests (incl. virology at screening)	X^b^	X	–	–	–	X	X	X	–	–	X	–	X^e^
Serum pregnancy test	X^c^	X^c^	–	–	–	X^c^	X^c^	X^c^	–	–	X^c^	–	–
Home urine pregnancy test	–	–	–	–	–	–	–	–	X^c^^,d^	X^c^^,d^	–	X^c^^,d^	–
Home body weight and ankle swelling self-check	–	–	X	X	X	–	–	–	X	X	–	X	–
Imatinib assay	–	X	–	–	–	X	X^i^	X^i^	–	–	X	–	–
Research blood samples	–	X	–	–	–	X	–	–	–	–	X	–	–
Electrocardiogram (ECG)	–	X	–	–	–	X	X	X	–	–	X	–	X^e^
Echocardiogram	X	–	–	–	–	–	–	–	–	–	X	–	X^e^
Brain MRI scan	X^a^	–	–	–	–	–	–	–	–	–	–	–	–
Optional CT head scan	X^a^^,f^	–	–	–	–	–	–	–	–	–	–	–	–
Quality of life questionnaire	–	X	–	–	–	X	–	–	–	–	X	–	–
Administration of Imatinib	–	X	–	–	–	–	–	–	–	–	X	–	–
Review of the diary, Imatinib collection and reconciliation	–	–	–	–	–	X	X	X	–	–	X	–	–
Report of adverse events, if any	–	X	X	X	X	X	X	X	X	X	X	X	X

*Except for patients with an implanted CardioMEMS™ device.

^a^Can be performed on a separate day ± 3 days apart from the original visit date, as needed.

^b^Virology tests apply to this visit only.

^c^For women of childbearing potential.

^d^Urine pregnancy (β-hCG) tests will be performed at home. A telephone call will be performed within 72 hours of the urine test date to enquire about the results.

^e^If clinically required.

^f^A CT head scan will be performed at screening, when MRI is contra-indicated or not tolerated. The brain MRI scan will always be the first choice.

^g^The follow-up telephone assessment can be performed after early termination as long as patients are off study drug for 4 weeks (±3 days).

^h^Can be omitted if patient had right heart catheterisation outside the study within a month prior to screening. On this occasion, retrospective catheterisation data (up to one month old) can be used for both screening and baseline entries.

^i^Subjects in Part 1 may undergo detailed pharmacokinetic sampling on one of these occasions, if not performed on Week 4.
